# Pressure Loading Induces DNA Damage in Human Hepatocyte Line L02 Cells via the ERK1/2–Dicer Signaling Pathway

**DOI:** 10.3390/ijms23105342

**Published:** 2022-05-10

**Authors:** Yanping Tang, Yanan Fan, Qing Luo, Guanbin Song

**Affiliations:** College of Bioengineering, Chongqing University, Chongqing 400030, China; 201919021048@cqu.edu.cn (Y.T.); fanyanan@cqu.edu.cn (Y.F.); qing.luo@cqu.edu.cn (Q.L.)

**Keywords:** compressive stress, hepatocyte, DNA damage, ERK1/2–Dicer signal

## Abstract

Alteration of liver tissue mechanical microenvironment is proven to be a key factor for causing hepatocyte injury and even triggering the occurrence of hepatocellular carcinoma; however, the underlying mechanisms involved are not fully understood. In this study, using a customized, pressure-loading device, we assess the effect of pressure loading on DNA damage in human hepatocytes. We show that pressure loading leads to DNA damage and S-phase arresting in the cell cycle, and activates the DNA damage response in hepatocytes. Meanwhile, pressure loading upregulates Dicer expression, and its silencing exacerbates pressure-induced DNA damage. Moreover, pressure loading also activates ERK1/2 signaling molecules. Blockage of ERK1/2 signaling inhibits pressure-upregulated Dicer expression and exacerbates DNA damage by suppressing DNA damage response in hepatocytes. Our findings demonstrate that compressive stress loading induces hepatocyte DNA damage through the ERK1/2–Dicer signaling pathway, which provides evidence for a better understanding of the link between the altered mechanical environment and liver diseases.

## 1. Introduction

Hepatocellular carcinoma (HCC) occurs mostly in cirrhotic livers and has a high incidence and mortality rate. Changes in the mechanics of the liver (solid stress, interstitial fluid pressure, and extracellular matrix stiffness) are linked to the development of hepatocellular carcinoma. Higher substrate stiffness significantly enhances the malignant phenotype of HepG2 in hepatocellular carcinoma cells by activating the Wnt/β–catenin pathway [1]. High substrate stiffness alters cell morphology, decreases hepatocyte-specific gene expression, and reduces hepatocyte-specific metabolism [2,3]. In addition, elevated shear stress has been shown to increase the severity of hepatocytes’ response to substrate stiffness, leading to dedifferentiation [3]. A growing number of studies have shown that mechanical forces can modulate the biological behavior of hepatocytes and lead to the malignant transformation of cells. Therefore, exploring the effects of mechanical force on hepatocytes may contribute to the discovery of new interventions to prevent the development of HCC.

Genomic instability is a feature of most cancers. Some recent studies have found that mechanical changes can lead to DNA damage in cells, and if this damage is not repaired, it can affect genomic stability and increase the possibility of carcinogenesis in normal cells. Chod et al. found that acute perturbation of compressive stress or ECM applied to actin leads to DNA damage in cardiac myocytes [4]. When DNA damage occurs, cells activate DNA damage response pathways that detect and repair this damage. Once the cell detects the lesion, it causes cell-cycle arrest and transcriptional repression to promote damage repair and prevent the propagation of mutation to daughter cells [5]. DNA damage and repair occur in the nucleus, and DNA repair factors are recruited to the area of damage when it occurs. Damage that is not repaired leads to cell death, through apoptosis, or to the propagation of mutations that drive genomic instability and cancer development [6,7]. Ataxia–telangiectasia-mutated protein (ATM) and DNA-dependent protein kinase catalytic subunit protein (DNA–PKC_S_) are the main initiators of DNA double-strand break repair [8]. ATM localizes to the damage region, where it phosphorylates histone H2AX to produce γ-H2AX. γ-H2AX then binds to DNA damage checkpoint protein 1 and MRN protein complexes at the break site, activating cyclin-8-mediated chromatin ubiquitination, recruiting 53BP1 to DNA damage sites for damage repair [9,10]. However, the effect of mechanistic factors on DNA damage in hepatocytes and the molecular mechanisms involved are not yet known.

Dicer is a type III cytoplasmic endonuclease that regulates the DNA damage response (DDR), a function that is not dependent on microRNA [11]. Rapidly dividing cells depend on Dicer to help them repair DNA damage caused by gene replication errors. In a mouse medulloblastoma model, Dicer deficiency was found to cause DNA damage and death in embryonic stem cells and cerebellar precursor cells [12]. After induction of DNA damage, Dicer generates small RNAs at the double-strand breaks (DSBs) loci, which are necessary for DDR [13]. Meanwhile, it has been reported that extracellular signal-regulated kinase 1/2 (ERK1/2) is involved in the DDR signaling pathway and regulates Dicer expression [14]. ERK1/2 has the ability to translocate to the nucleus and phosphorylate transcription factors, and activation of the ERK signaling pathway during DDR contributes to the activation of the DDR checkpoint, which inhibits cytokinesis [15]. In addition, it has been shown that mechanical factors can activate ERK1/2 and, thus, regulate cell behavior [16]. Therefore, we hypothesized that mechanical forces regulate Dicer by affecting the activation of ERK1/2.

In this study, we investigated the DNA damage of normal liver cells in response to stress, and the results further revealed the possible molecular mechanisms involved. We found that higher stress drives increased the expression level of Dicer, as well as DNA damage accumulation, possibly through the ERK1/2–Dicer signaling pathway.

## 2. Results

### 2.1. Pressure Aggravates DNA Damage in Hepatocytes and Activates the DDR Pathway

**Figure 1:**
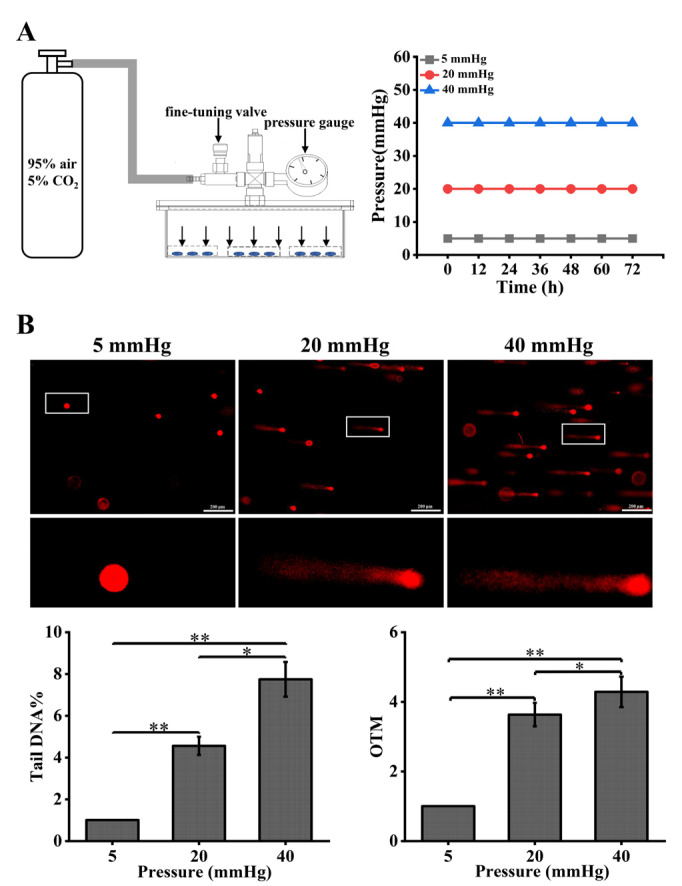
Pressure loading aggravates DNA damage in hepatocytes: (**A**) schematic diagram of the experimental apparatus for pressure and changes in pressure over time in the pressure loading device; (**B**) DNA damage in hepatocytes cultured for 48 h under different pressure levels (5, 20, 40 mmHg) was examined using the comet assay. OTM and tail DNA% were used to calculate the degree of DNA damage (scale bar, 200 µm). Data are expressed as the mean ± SD. *n* = 3, * *p* < 0.05, ** *p* < 0.01.

Studies have found that mechanical stimuli can lead to DNA damage in some types of cells [17], but DNA damage in hepatocytes under pressure has not been clearly demonstrated. To study the effect of pressure on DNA damage in hepatocytes, we designed and fabricated an in vitro pressure loading device (Figure 1A) for simulating normal portal pressure (5 mmHg) and portal hypertension (20 mmHg and 40 mmHg). A gas mixture was introduced to increase the internal pressure. While introducing the gas, no prepacked room air was released, so the partial pressure of the gas contained in the device remained constant according to the Boyle–Charles law. After 48 h of incubation, the comet assay was used to determine the extent of DNA damage in hepatocytes. We found that the trailing phenomenon was more pronounced in hepatocytes under two high pressures compared with 5 mmHg, and the DNA damage indicators Tail DNA% and OTM values were significantly increased (Figure 1B). The results indicated that the degree of DNA damage in hepatocytes increased significantly with increasing pressure.

**Figure 2:**
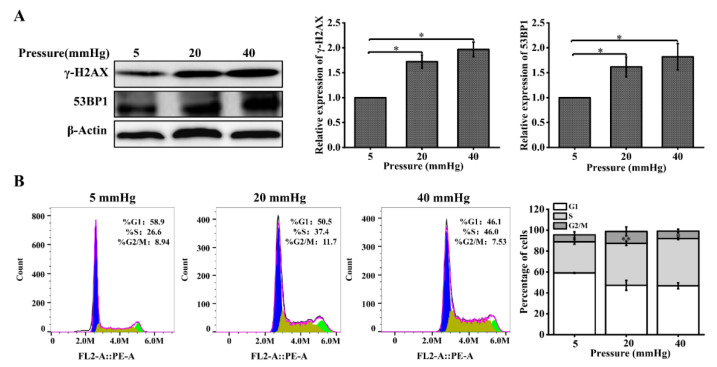
Pressure loading results in increased expression of DNA repair proteins and cell-cycle arrest in hepatocytes: (**A**) DNA repair proteins in hepatocytes were examined with Western blot and quantification data of the expression levels of γ-H2AX and 53BP. The cells were exposed to pressure for 48 h; (**B**) cell-cycle distribution was analyzed using flow cytometry following pressure loading for 48 h. The images are representative of three independent experiments. The stacked bar graph indicates the percentage of cells at various stages of the cell cycle. Data are expressed as the mean ± SD. *n* = 3, * *p* < 0.05, ** *p* < 0.01.

Cells undergoing DNA damage initiate the DDR signaling pathway in response to DNA damage, and this pathway can respond to DNA damage through cell-cycle arrest, activation of DNA repair pathways, and induction of apoptosis [18,19,20]. First, we examined the changes in the expression of the DNA repair proteins γ-H2AX and 53BP1 with Western blot. The protein expression levels of γ-H2AX and 53BP1 were significantly increased in hepatocytes cultured under higher pressure, compared with 5 mmHg (Figure 2A). Flow cytometry was used to determine the proportional changes in the cycle distribution of hepatocytes under the effect of pressure. We found that pressure changed the percentage of hepatocytes in different cell-cycle phases. The percentage of S-phase cells was 22.6%, 37.4%, and 46% after 48 h of incubation under pressure, and higher pressure significantly increased the percentage of S-phase cells, compared with a pressure of 5 mmHg (Figure 2B). In conclusion, these results suggest that higher stress induces DNA damage in hepatocytes, and the cells respond to this change by increasing the expression of DNA repair proteins and arresting the cell cycle.

### 2.2. Downregulation of Dicer Expression Exacerbates Stress-Induced DNA Damage by Attenuating the DDR Pathway Response

**Figure 3:**
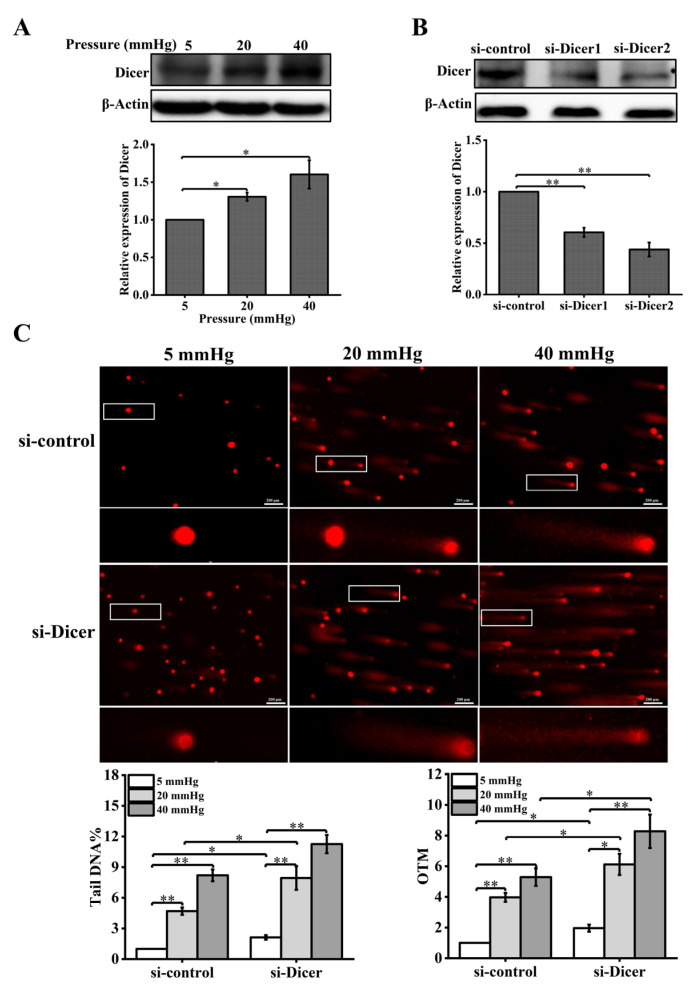
Reduced Dicer expression levels enhance pressure-induced DNA damage in hepatocytes: (**A**) hepatocytes were treated with different pressure levels (5, 20, 40 mmHg) for 48 h. Western blot analysis showed the protein level of Dicer; (**B**) the cells were transfected with negative control siRNA or Dicer siRNAs for 48 h. Western blot showed the protein level of Dicer after knockdown of Dicer; (**C**) DNA damage in hepatocytes cultured for 48 h under different pressure levels (5, 20, 40 mmHg) with Dicer knockdown was examined with the comet assay. OTM and tail DNA% were used to calculate the degree of DNA damage (scale bar, 200 µm). Data are expressed as the mean ± SD. *n* = 3, * *p* < 0.05, ** *p* < 0.01.

In the presence of exogenous DNA damage, Dicer produces small non-coding RNAs that are required for DDR [21]. To test whether Dicer is regulated by pressure, we examined the expression of Dicer in hepatocytes treated with different pressure levels. The expression level of Dicer was measured with Western blot and was found to be upregulated with increased pressure (Figure 3A). To determine the effect of Dicer on stress-induced DNA damage, we knocked down Dicer using specific siRNA and detected the expression level of Dicer using Western blot analysis. The results showed that the expression of Dicer was significantly inhibited (Figure 3B). In addition, knockdown of Dicer resulted in enhanced DNA damage in pressure-stimulated hepatocytes (Figure 3C).

**Figure 4:**
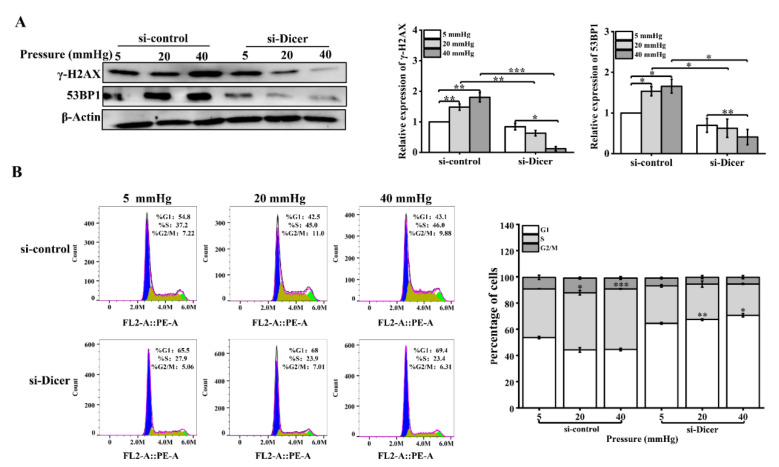
Reduced Dicer expression causes downregulation of DNA repair protein expression and cell-cycle changes in stress-loaded hepatocytes: (**A**) DNA repair proteins in hepatocytes with Dicer knockdown were examined with Western blot and quantification data of the γ-H2AX and 53BP1 protein levels. Cells were exposed to pressure for 48 h; (**B**) cell-cycle distribution was analyzed using flow cytometry following Dicer knockdown and pressure loading for 48 h. The images are representative of three independent experiments. The stacked bar graph indicates the percentage of cells at various stages of the cell cycle. Data are expressed as the mean ± SD. *n* = 3, * *p* < 0.05, ** *p* < 0.01, *** *p* < 0.001.

Meanwhile, we examined the expression levels of γ-H2AX and 53BP1 and the cell-cycle distribution changes after Dicer knockdown. We found that the expression levels of γ-H2AX and 53BP1 were significantly downregulated in hepatocytes with Dicer knockdown under pressure (Figure 4A), and the knockdown of Dicer in hepatocytes resulted in a lower percentage of S-phase cells and an increased number of G1-phase cells (Figure 4B). Overall, Dicer may lead to increased DNA damage in hepatocytes under pressure by inhibiting DNA damage repair and affecting cell-cycle progression.

### 2.3. ERK1/2 Signaling Molecules Regulate Dicer Expression Levels and DNA Damage in Hepatocytes under Pressure Loading

**Figure 5:**
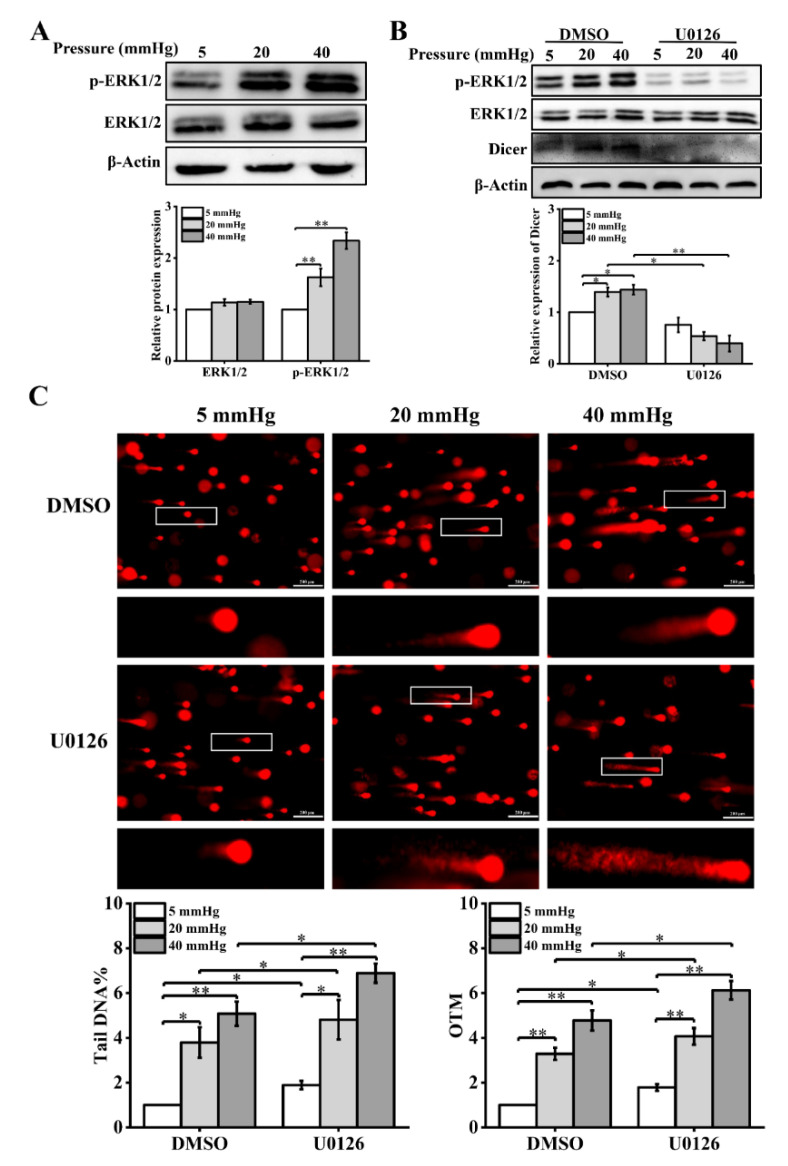
Pressure activates ERK1/2, and U0126 treatment leads to increased DNA damage in hepatocytes under pressure: (**A**) Western blots showing the protein levels of p-ERK1/2 and ERK1/2 in hepatocytes treated with pressure for 48 h. Histogram showing the mean level of total ERK1/2 and ERK1/2 activation (p-ERK1/2/total ERK1/2); (**B**) Western blots show the protein levels of p-ERK1/2, ERK1/2, and Dicer in hepatocytes treated with U0126 (10 μM). Histogram shows mean level of Dicer expression from three independent experiments; (**C**) DNA damage in U0126-treated hepatocytes cultured for 48 h under different pressure levels (5, 20, 40 mmHg) was examined with the comet assay. OTM and tail DNA% were used to calculate the degree of DNA damage (scale bar, 200 µm). Data are expressed as the mean ± SD. *n* = 3, * *p* < 0.05, ** *p* < 0.01.

ERK1/2 regulates a variety of cellular biological processes in response to stress under normal and pathological conditions, such as proliferation, differentiation, apoptosis, and key signaling pathways [22,23]. Phosphorylated ERK1/2 (p-ERK1/2) has been reported to be altered in response to mechanical stimulation [24]. In addition, p-ERK1/2 regulates the transcriptional level of Dicer [14]. Therefore, we speculated that stress might affect Dicer expression levels through ERK1/2 signaling molecules. First, we examined the expression of ERK1/2 and its phosphorylation level by Western blot. The phosphorylation level of ERK1/2 was found to be significantly increased under higher stress (Figure 5A). Then, to explore the effect of ERK1/2 signaling molecules on Dicer expression, hepatocytes under different stress loading conditions were treated with a MEK1/2 inhibitor (U0126). The expression of p-ERK1/2 was significantly inhibited after U0126 treatment and U0126 decreased the Dicer expression level induced by higher pressure (Figure 5B). Later, we found that U0126 treatment exacerbated DNA damage in hepatocytes cultured under pressure by the results of the comet assay (Figure 5C).

**Figure 6:**
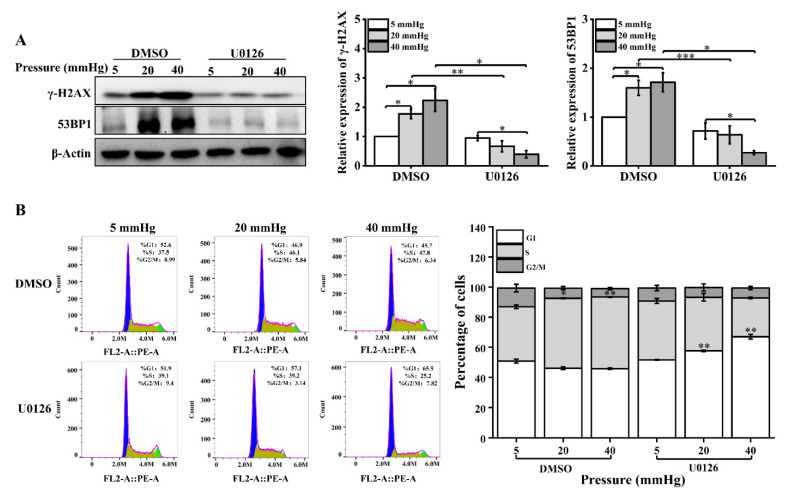
U0126 treatment causes downregulation of DNA repair proteins and cell-cycle changes in pressure-loaded hepatocytes: (**A**) DNA repair proteins in hepatocytes treated with U0126 (10 μM) were examined with Western blot and quantification data of the γ-H2AX and 53BP1 protein levels. The cells were exposed to pressure for 48 h; (**B**) cell-cycle distribution was analyzed using flow cytometry following U0126 treatment and pressure loading for 48 h. The images are representative of three independent experiments. The stacked bar graph indicates the percentage of cells at various stages of the cell cycle. Data are expressed as the mean ± SD. *n* = 3, * *p* < 0.05, ** *p* < 0.01, *** *p* < 0.001.

In addition, we examined the changes in the expression levels of γ-H2AX and 53BP1 and cell-cycle distribution after U0126 treatment. The expression of γ-H2AX and 53BP1 was significantly downregulated in pressure-loaded hepatocytes treated with U0126 (Figure 6A). The distribution ratio of each phase of the cell cycle was significantly altered, showing that the inhibition of ERK1/2 activation decreased the proportion of S-phase cells and increased the number of G1-phase cells (Figure 6B). In conclusion, these results suggest that the expression of Dicer induced by stress was influenced by the activation of ERK1/2. Additionally, the extent of DNA damage induced by pressure in hepatocytes was regulated by the recruitment of DNA repair proteins and cell-cycle arrest through the ERK1/2–Dicer signaling pathway.

## 3. Discussion

In this study, we administered varying degrees of mechanical pressure to hepatocytes within a pressure-loading device to simulate portal hypertension due to cirrhosis. The pathophysiological process of portal hypertension occurs in different circulatory compartments: intrahepatic circulation, visceral circulation, and collateral circulation [25]. Portal pressure is the product of intrahepatic vascular resistance and portal blood flow through the hepatic vessels. The presence of portal hypertension increases the pressure in the sinusoids, causing mechanical stress on liver cells [26]. We found that pressure aggravated DNA damage and regulated DNA damage repair and cell-cycle progression in hepatocytes through the ERK1/2–Dicer signaling pathway, which, in turn, affected DNA damage.

It is well known that genetic instability is an important hallmark of tumorigenesis, mainly due to DDR deficiency and increased replication stress. As the major cell population in the liver, hepatocytes are essential to maintain liver function homeostasis. During cirrhosis, cells have highly irregular nuclear morphologies, and nuclear deformation increases genetic instability and the accumulation of DNA damage in cells [27,28,29]. Numerous studies have shown that, after a long evolutionary period, cells have developed multiple pathways to cope with DNA damage, the most important mechanism of which is the DDR signaling pathway, which can respond to DNA damage through cell-cycle arrest, activation of DNA repair pathways, and induction of apoptosis. Here, we found increased DNA damage, increased expression of DNA repair proteins, and cell-cycle arrest in S phase in hepatocytes under stress.

Studies have reported that Dicer is involved in the DNA damage repair process in cells [11,30]. We found that the expression level of Dicer was increased under high stress. Similar to our results, Zhang et al. used three chemical and physical methods—cisplatin, Adriamycin, and ionizing radiation—to cause DNA damage in cells, and Western blot assays revealed upregulated expression levels of Dicer [31]. DNA damage generated using I-SceI cleavage has also been shown to lead to elevated Dicer expression [32]. Moreover, we detected that stressed knockdown Dicer hepatocytes exhibited more pronounced DNA damage and that the expression levels of DNA repair proteins γ-H2AX and 53BP1 were significantly reduced. Francia et al. showed that Dicer inactivation significantly reduced the number of positive cells for DDR foci containing 53BP1, the autophosphorylated form of ATM, and phosphorylated substrates of ATM and ATR, and no significant reduction occurred in the number of positive cells expressing γ-H2AX [33]. Flow cytometry examined the cell-cycle distribution of hepatocytes with knockdown of Dicer under stress conditions and found that the cells were arrested in the G1 phase. Liu et al. found that knockdown of Dicer resulted in prolonged cell transition from G1 to S phase, consistent with our results [34]. As a key player in the DNA damage response, Dicer can link mechanical stimuli to DNA damage in hepatocytes.

Accumulating evidence has implicated the MEK/ERK signaling pathway in the DNA damage response in cells [15]. During DDR, the MEK/ERK pathway is activated and is involved in activating the DDR checkpoint and causing cell-cycle arrest [35]. In our study, it was found that the expression levels of p-ERK1/2 increased with higher pressure, indicating that ERK1/2 signaling molecules were activated under higher stress. Similar to our findings, it was found that ERK activity was significantly increased and regulated osteogenic differentiation in mesenchymal stem cells cultured on hard hydrogels [36]. Moreover, Liu et al. found that stiffer hydrogels resulted in increased ERK1/2 activity in HCC cells, which, in turn, mediated YAP activation in HCC cells [24]. These findings suggest that the activation of ERK1/2 signaling molecules is affected by external mechanical stimuli. One study has reported that ERK1/2 is involved in gemcitabine resistance in pancreatic cancer cells by promoting the transcriptional level of Dicer [14]. Furthermore, it was found that in etoposide-induced G2/M phase block in MCF7 cells, knockdown of ERK1/2 significantly reduced the response of ATM to etoposide treatment, thereby attenuating the level of H2AX phosphorylation and impairing etoposide-induced cell-cycle arrest [37]. Our results showed that the expression level of p-ERK1/2 was increased under high-stress conditions. It was found that inhibition of ERK1/2 activity under high stress can downregulate Dicer expression, increase the extent of DNA damage, downregulate the expression of DNA repair proteins, and stall cell-cycle progression. Our results provide a mechanistic link by which ERK1/2 can respond to stress-induced DNA damage by regulating the expression of Dicer.

**Figure 7:**
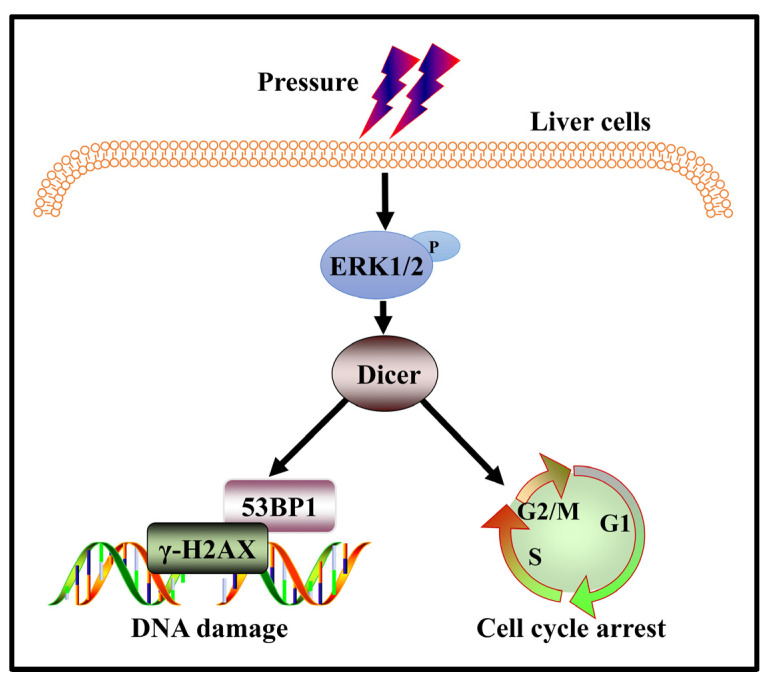
Schematic model of DNA damage induced by higher pressure in hepatocytes. Higher stress results in aggravated DNA damage in hepatocytes. Higher pressure activates ERK1/2 and upregulates the expression level of Dicer. Then, Dicer mediates the DDR of the cell. After Dicer expression downregulation, the DDR function of the cells is diminished, and the DNA damage of the cells is enhanced in response to stress.

In conclusion, our study provides an important link between the ERK1/2–Dicer signaling pathway and the accumulation of DNA damage in hepatocytes under stress (Figure 7). We showed that the downregulation of Dicer expression results in increased DNA damage and/or reduced DNA repair capacity in cells subjected to stress. Stress changes lead to cell-cycle arrest and DSB accumulation, which are factors contributing to genomic instability. Therefore, Dicer could be a potential target for intervention in hepatocyte DNA damage during cirrhosis. However, detailed mechanisms regarding the role of Dicer in response to stress-induced DNA damage in hepatocytes remain to be investigated.

## 4. Materials and Methods

### 4.1. Cell Culture

The normal human L02 liver cell line was purchased from the Shanghai Institute of Biochemistry and Cell Biology, Chinese Academy of Sciences. The liver cells were cultured in an RPMI-1640 medium (Gibco, New York, MA, USA) supplemented with 10% (*v*/*v*) fetal bovine serum (HyClone, Logan, UT, USA) and antibiotic supplement (100 U/mL penicillin and 100 μg/mL streptomycin) at 37 °C in a humidified incubator with 95% air and 5% CO_2_. 

### 4.2. Pressure-Loading Apparatus and Pressure Loading on Cells

The pressure-loading device (Figure 1A) consists of a stainless-steel box in which cell culture plates are placed and a pressure control system. The pressure control system comprises a gas pipe, a fine-tuning needle valve, a safety valve, and a pressure gauge. A mixture of 95% air and 5% CO_2_ was inflated from the inlet tube to increase the internal pressure. The pressure required for the experiment was obtained by adjusting the fine-tuning valve and the pressure gauge. Normal portal venous pressure (5 mmHg) was selected as the control condition, and the pressure values to simulate cirrhotic hypertension were set at 20 mmHg and 40 mmHg. The L02 cells were spread in a 6-well plate (Corning, New York, NY, USA) at a density of 2 × 10^5^ cells/well, 2 mL of complete medium was added, and the cells were incubated overnight. Next, the culture plate was placed into the cassette by removing the upper part, and the device was resealed with screws. Gas was charged into the device from the inlet tube by regulating the fine-tuning valve.

A mixture (95% air and 5% CO_2_) was injected into the pressure loading device so that the pressure values were 5, 20, and 40 mmHg, and the values on the pressure gauge were observed and recorded at 0 h, 1 h, 12 h, 24 h, 36 h, 48 h, 60 h, and 72 h, respectively. After 1 h, the pressure values rose due to the temperature inside the incubator being higher than the temperature of the pressure device itself, at which point, the pressure values were adjusted to the initial setting and the pressure values at subsequent time points were maintained at 5, 20, and 40 mmHg, indicating that the device was well sealed and could be used for subsequent experiments (Figure 1A).

### 4.3. Comet Assay

After pressure-loading treatment, hepatocytes in the treatment group were washed, trypsinized, and suspended in ice-cold PBS at a cell concentration of 3 × 10^5^ cells/mL. A volume of 50  μL cell suspension, combined with 100 μL low-melting-point agarose (0.6% (*w*/*v*), was pipetted onto a 0.8% (*w*/*v*) agarose-coated frosted glass slide, covered with a coverslip and placed for 10  min at 4  °C. Then, the coverslip was gently removed, and the slide was immersed in prechilled lysis solution (100 mM Na2EDTA, 2.5 M NaCl, 10 mM Tris-HCl with 10% DMSO and 1% Triton X-100 freshly added, pH 10) for 2 h at 4 °C. After lysis, the slide was washed with prechilled electrophoresis, incubated in electrophoresis for 20 min, and then subjected to electrophoresis at 20 V for 30  min at 4  °C. The slide was neutralized with PBS, dried with absolute ethanol, and stained with GelRed Nucleic Acid Staining (Beyotime Biotechnology, Shanghai, China). Cell images were captured with a fluorescent microscope (Olympus, Tokyo, Japan). CASP software was used to analyze these images. The percentage of DNA in the tail and the Olive tail moment were measured to determine the level of DNA damage.

### 4.4. Cell-Cycle Analysis

For cell-cycle analysis, the treated hepatocytes were digested and washed with ice-cold PBS. Then, the cells were centrifuged at 2000× *g* for 5 min to obtain cell pellets. The supernatant was discarded, and 50 μL of liquid was saved to disperse the cells. Cells were mixed with 1 mL of precooled 70% ethanol at 4 °C overnight. The cells were collected by centrifugation and washed once with PBS. Finally, the cells were stained with a 500 μL PI working solution at 37 °C in the dark for 30 min. The stained cells were detected using a CytoFLEX flow cytometer (Beckman Coulter, Boston, MA, USA), and data were collected using FlowJo version 10.0.7 software (TreeStar, San Carlos, CA, USA).

### 4.5. siRNA Transfection

The siRNAs targeting human Dicer1 (siRNA#1: CAGGAACAUAUCAGAUUUATT; siRNA#2: GGACCAUUUACUGACAGAATT) and negative control siRNA were purchased from Genepharma (Shanghai, China). For Dicer knockdown with siRNA, siRNAs were transiently transfected into hepatocytes using Lipofectamine 3000 (Invitrogen, Thermo Fisher Scientific, New York, MA, USA), following the manufacturer’s instructions. 

### 4.6. Western Blot Analysis

After washing with cold PBS, the total protein from liver cells was isolated with ice-cold RIPA lysis buffer (Beyotime, Shanghai, China) containing a 1% protease inhibitor cocktail (Bimake, San Francisco, CA, USA). Protein concentration was quantified by the BCA protein assay kit (Beyotime, Shanghai, China). Briefly, 5× SDS loading buffer was added to each protein sample, mixed well, and boiled for 10 min for Western blot analysis. Protein samples (30 µg) were fractionated using 10% SDS–PAGE gel and transferred to activated PVDF membranes (Millipore, Billerica, MA, USA). The membranes were blocked with 5% non-fat milk in Tris-buffered saline (TBS) with 0.05% Tween 20 (TBST) for 1 h at room temperature. Membranes were then incubated overnight at 4 °C with primary antibodies (β-Actin, Zhongshan Golden Bridge Biotechnology, TA-09, Beijing, China), (t-ERK1/2, Abcam, ab184699, Cambridge, UK), (p-ERK1/2, Cell Signaling Technology, 4370T, Danvers, MA, USA), (γ-H2AX, Abcam, ab81299, Cambridge, UK), (53BP1, Abcam, ab175933, Cambridge, UK), and (Dicer, Abcam, ab16744, Cambridge, UK). Afterward, membranes were washed 3 times in TBST for 10 min and then incubated with the HRP-conjugated secondary antibodies (ZENBIO, Chengdu, China) for 1 h at room temperature with shaking, followed by 3 rounds of 10 min wash in TBST. Protein signals were detected with an ECL blotting analysis system (Bio-OI, Guangzhou, China).

### 4.7. Statistical Analysis

All presented data and results were confirmed by at least three replicates per condition. The results are presented as the means ± SD. Data were compared using a one-way analysis of variance, followed by Student’s *t*-test. Statistical significances were accepted at *p* < 0.05.

## Data Availability

All data are contained within the article.
